# Do Patient Sex and Age Affect Hemiepiphysiodesis Outcomes?

**DOI:** 10.3390/jcm13061654

**Published:** 2024-03-14

**Authors:** Piotr Morasiewicz, Paweł Leyko, Łukasz Tomczyk, Krystian Kazubski

**Affiliations:** 1 Department of Orthopaedic and Trauma Surgery, Institute of Medical Sciences, University of Opole, Witosa 26, 45-401 Opole, Poland; 2 Department of Food Safety and Quality Management, Poznan University of Life Sciences, Wojska Polskiego 28, 60-637 Poznan, Poland

**Keywords:** hemiepiphysiodesis, valgus knee, varus knee, O-Plate, sex, age, radiological results, clinical results

## Abstract

(1) **Background**: The purpose of this study was to assess the effects of sex and age on the outcomes of hemiepiphysiodesis performed for genu valgum and varum deformity correction. (2) **Methods**: We analyzed patients who had undergone O-Plate hemiepiphysiodesis due to genu valgum or varum in the period of 2020–2023. The study group comprised 22 females and 20 males aged between 3 and 14 years at the time of surgery. Age-stratification yielded a subgroup of 3–10-year-olds (16 patients, 20 treated limbs) and a subgroup of 11–14-year-olds (26 patients, 28 treated limbs). We assessed the following parameters: hospital stay duration, deformity correction time, MAD correction, amount of angular correction, correction velocity, correction rate, complete deformity correction, deformity recurrence, surgery duration, and complications. (3) **Results**: The mean follow-up was 19 months. The mean surgery time in the subgroup of 3–10-year-olds (25.62 min) was significantly longer than that in the subgroup of 11–14-year-olds (22.81 min, *p* = 0.018). The mean deformity correction time in the male subgroup (11.33 months) was significantly shorter than that in the female subgroup (15.87 months, *p* = 0.013). A comparison of the subgroups stratified by age yielded a mean amount of angular correction of 10.5° in the younger children, which was significantly higher than that of 7.2° achieved in the older children; *p* = 0.027. The difference in mean correction velocity between 3–10-year-old children (4.03 mm/month) and that in 11–14-year-old children (1.39 mm/month) was statistically significant; *p* = 0.031. The mean rate of correction was 0.49°/month in females and 0.89°/month in males, with the latter rate significantly greater; *p* = 0.023. The difference in the mean rate of correction between the younger (1.08°/month) and the older subgroup (0.59°/month) was also significant; *p* = 0.018. A significant difference in terms of deformity recurrence rates was observed between the younger subgroup (66.67%) and older subgroup (only 10.53%); *p* = 0.005. (4) **Conclusions**: Patient sex had no significant effect on hemiepiphysiodesis outcomes; patient age has a considerable effect on hemiepiphysiodesis outcomes.

## 1. Introduction

Pediatric lower limb deformities are a considerable problem for orthopedic surgeons [1,2,3,4,5,6,7,8,9,10,11,12,13,14,15,16,17,18,19,20,21,22,23,24]. Genu valgum or varum may cause limping, pain, knee or patellar instability, gait disturbances, limited mobility, articular cartilage damage, accelerated joint degeneration, rapid fatigue, sports and physical activity limitations, meniscal injury, and cosmetic concerns [1,2,3,4,5,6,7,8,9,10,11,12,13,14,15,16,17,18,24]. Genu valgum or varum deformity treatment is indicated in cases with a mechanical axis deviation (MAD) of >10 mm or the inter-malleolar distance of >8 cm, or an angular deformity of >10° [1,3,7,11,12,14,16]. Since conservative treatment methods (rehabilitation, exercises, insoles, orthoses, plaster casts) for pediatric lower limb deformities are ineffective, surgical treatment is recommended in this patient population [1,2,3,4,5,6,7,8,9,10,11,12,13,14,15,17,22,23]. The techniques of pediatric lower limb deformity correction include the Ilizarov method osteotomy, osteotomy with plate fixation, osteotomy with external fixation, and hemiepiphysiodesis with the use of stapling or an O-plate (also known as an eight-plate) [1,2,3,4,5,6,7,8,9,10,11,12,13,14,15,16,17,18,19,20,21,22,23,24]. Hemiepiphysiodesis is indicated for smaller deformities (less than 11 degrees) and in patients aged approximately 0.5–2 years prior to the predicted growth plate fusion or at an age of 14–16 years [6,7,8,10,11,14]. Due to the limitations and lower effectiveness of hemiepiphysiodesis, in the case of larger deformities (exceeding 12 degrees) and in older patients over 14–16 years of age, it is recommended to perform osteotomy with plate fixation or osteotomy with external fixation [6,7,8,10,11,14]. Hemiepiphysiodesis is a minimally invasive, quick, inexpensive method with good treatment outcomes and low complication rates; therefore, it has been widely adopted worldwide [1,2,3,4,6,7,8,9,10,11,12,13,14,15,18,21,22].

Little is known of the potential effects of patient sex and age on hemiepiphysiodesis outcomes. Previous research focused on the clinical and radiological outcomes of hemiepiphysiodesis [1,2,3,4,9,10,11,12,13,18,19,21,22,23,24]; other authors compared the outcomes of this procedure depending on the implant type used [6,8,17,20]. Some literature reports suggest that effective hemiepiphysiodesis that ensures complete deformity correction requires the procedure to be performed approximately 0.5–2 years prior to the predicted growth plate fusion or at an age of 14–16 years, at the latest [6,7,10,14]. Other authors suggest that to be the most effective, hemiepiphysiodesis should be performed under the age of 10 years [2]. On the other hand, the risk of deformity recurrence is higher in patients under 10 years of age [7]. The greater risk of recurrence of the deformity after implant removal in younger children is due to the presence of still-open growth plates. Raab reports a higher risk of complications and recurrence of deformity in younger patients [11]. Patients under 10 years of age may have a higher correction rate but also a higher risk of rebound [4,7,10]. Burghardt et al. deem it unwise to delay hemiepiphysiodesis in small children with deformities until they are close to adolescence [22]. Radtke et al. assert that it is important to assess the effect of patient age on hemiepiphysiodesis outcomes [12]. However, there have been no studies evaluating the effect of age on the outcomes of O-plate hemiepiphysiodesis and deformity correction. Theoretically, younger children, whose growth is more rapid, should have better hemiepiphysiodesis outcomes than older children. Growth in girls and boys occurs with different dynamics and intensifies at different years of life [25,26], which may theoretically influence the dynamics of deformity correction and the results after hemiepiphysiodesis. In girls, the period of intensive growth (growth spurt) occurs faster than in boys [25,26]. In boys, the period of intensive growth appears almost 2 years later and is more intense than in girls [25,26]. The effects of patient sex on hemiepiphysiodesis outcomes have, likewise, not been assessed. There are no methods that help predict the extent of angular deformity progression in pediatric patients [14].

We posed a hypothesis that patient sex and age affect hemiepiphysiodesis outcomes.

The purpose of this study was to assess the effects of sex and age on the outcomes of hemiepiphysiodesis performed for genu valgum and varum deformity correction.

## 2. Materials and Methods

This study was retrospective in nature. We analyzed patients who had undergone O-Plate PediPlates (OrthoPediatrics, Warsaw, IN, USA) hemiepiphysiodesis of the distal femur or proximal tibia due to genu valgum or varum in the period 2020–2023.

The study inclusion criteria were hemiepiphysiodesis of the distal femur or proximal tibia for genu valgum or varum deformity correction, complete medical and radiological records, informed consent, absence of other lower limb pathology, and a follow-up period of over 12 months after implant removal. The exclusion criteria were incomplete medical or radiological records, lack of informed consent, lower limb comorbidities, a follow-up period of <12 months, metabolic conditions, post-traumatic deformities, neuromuscular disorders, and post-inflammatory deformities. The study was approved by the local ethics committee.

Once the inclusion and exclusion criteria were applied, 42 patients (48 treated limbs) remained for analysis. The study group comprised 22 females and 20 males aged between 3 and 14 years at the time of surgery. For the sake of comparison, the study population was stratified by sex and age. Sex-stratification yielded 22 females (26 treated limbs) and 20 males (22 treated limbs), and age-stratification yielded a subgroup of 3–10-year-olds (16 patients, 20 treated limbs) and a subgroup of 11–14-year-olds (26 patients, 28 treated limbs).

**Figure 1:**
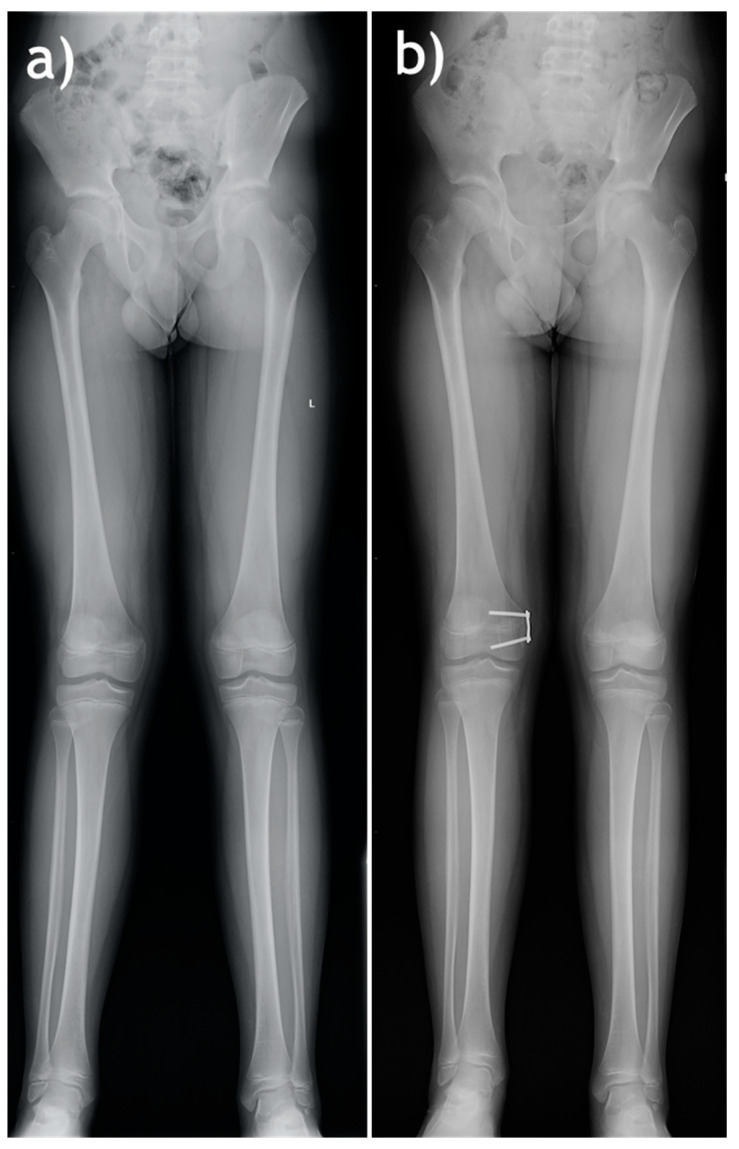
A sample patient with genu valgum—preoperative (**a**) and follow-up after hemiepiphysiodesis (prior to implant removal) X-ray images (**b**).

Each surgical procedure was conducted by one of two experienced orthopedic surgeons. All hemiepiphysiodesis procedures were conducted in children with open growth plates, with the use of O-Plate PediPlates implants (OrthoPediatrics, Warsaw, IN, USA) comprising two cannulated screws and a plate with two screw holes, Figure 1.

Indications for treatment were confirmed based on a clinical examination and radiographic evidence of deformity. The radiographic assessments were conducted by a single individual based on full-weight-bearing long-standing radiographs, obtained preoperatively and during periodic outpatient follow-up visits. The evaluated parameters were mechanical axis deviation (MAD) expressed in millimeters and defined as the distance from the mechanical axis of the lower limb to the center of the knee joint [1,12,15,16,19,21]; the mechanical medial proximal tibial angle (mMPTA); and the mechanical lateral distal femoral angle (mLDFA) [1,2,4,6,12,15,16,19,21]. Hemiepiphysiodesis was indicated by an MAD of >10 mm, radiographic evidence of deformity progression or at least a lack of deformity regression over three months, and an age of 1–2 years prior to the predicted end of bone growth [6,12,16]. Implant placement in the proximal tibia or the distal femur was determined by the location of a primary deformity assessed based on abnormal mMPTA and mLDFA values [6,12]. All procedures were performed under general anesthesia, with the patient in a supine position. The position of the growth plate and precise implant placement were ascertained under fluoroscopy. A 1–3 cm skin incision was made, followed by centering an O-Plate PediPlates implant about the physis under fluoroscopy. On postoperative day 1, patients were encouraged to walk with two elbow crutches bearing full weight on the operated limb. Follow-up visits took place in an outpatient setting every three months and involved a radiographic assessment of the lower limbs. The implants were not removed until complete deformity correction was achieved or until growth plates closed completely. If complete deformity correction was achieved before growth plate fusion, the implant was left in until growth plate fusion or until a slight 3–5-degree overcorrection, or an MAD of 2 mm, was achieved [1,2,6,7,14,22]; radiographic follow-up assessments were conducted every 1–2 months.

In this study, we assessed the following parameters: hospital stay duration, deformity correction time, MAD correction, amount of angular correction, correction velocity, correction rate, complete deformity correction, deformity recurrence, surgery duration, and complications. All parameters were assessed based on the available medical and radiological records.

The duration of hospital stay was measured in days. Deformity correction time was defined as the time from implant insertion to implant removal and was expressed in months [1,4,6,9,12,18]. The amount of MAD correction was defined as the change in MAD from baseline to implant removal and expressed in millimeters [1,12]. The amount of angular correction (expressed in degrees) was defined differently for tibial and femoral implants. In the case of proximal tibial implants, this parameter was defined as the difference between the preoperative mMPTA and mMPTA at the time of implant removal. In the case of distal femoral hemiepiphysiodesis, this parameter was defined as the difference between the preoperative mLDFA and the mLDFA at the time of implant removal [8,11]. Correction velocity was defined as the ratio between the amount of MAD correction (millimeters) and implant indwelling time (months) [1,12,19]. The rate of correction was defined as the ratio of the amount of angular correction (mMPTA or mLDFA, expressed in degrees) and implant indwelling time (months) [4,6,17,18,19]. Complete deformity correction was defined as an MAD of ≤1 mm at the time of implant removal. Deformity recurrence was defined as a recurrence of the angular deformity (mMPTA or mLDFA of more than 3° in comparison with normal values) in the most recent follow-up radiographic assessment or the recurrence of an intercondylar or intermalleolar distance of >5 cm [19]. Surgery duration was measured from the beginning to the end of the procedure and expressed in minutes.

Complications were assessed based on the available medical and radiological records and included implant breakage, implant loosening, the necessity of revision surgery, delayed wound healing, edema, infections, vascular damage, nerve injury, soft tissue necrosis, limited range of motion, persistent pain, and overcorrection (defined as a hypercorrected MAD of >2 mm at a long-term follow-up). Monitoring and identification of complications were carried out based on the analysis of medical and radiological records as well as clinical examination during the hospital stay and periodic outpatient follow-ups.

All outcomes were assessed in the subgroups stratified by sex (males vs. females) and age (3–10 years vs. 11–14 years). All results/outcomes were validated between three observers.

### Statistical Analysis

Data were statistically analyzed using Statistica 13.1. The Shapiro–Wilk test was used to check for normality of the distribution. The Mann–Whitney U test was used to compare quantitative variables. The Pearson’s chi-squared test was used to compare quantitative variables. The level of statistical significance was set at *p* < 0.05.

## 3. Results

The mean follow-up was 19 months (ranging from 12 to 31 months). Detailed results are presented in Table 1 and Table 2.

The duration of hospital stay was three days in each subgroup.

The mean surgery duration was 23.8 min in the female subgroup and 23.7 min in the male subgroup, with the difference not statistically significant. However, the mean surgery time in the subgroup of 3–10-year-olds (25.62 min) was significantly longer than that in the subgroup of 11–14-year-olds (22.81 min, *p* = 0.018).

The mean deformity correction time in the male subgroup (11.33 months) was significantly shorter than that in the female subgroup (15.87 months, *p* = 0.013). The mean deformity correction time in 3–10-year-olds was 10.12 months and that in 11–14-year-olds was 13.91 months, with the difference not statistically significant.

The mean amount of MAD correction was 21.84 mm in the male group and 16.72 mm in the female group; and 38 mm in 3–10-year-olds and 15.8 mm in 11–14-year-olds. Neither difference was statistically significant.

The mean amount of angular correction was 8.53° in the male subgroup and 6.81° in the female subgroup, with the difference not statistically significant. A comparison of the subgroups stratified by age yielded the mean amount of angular correction of 10.5° in the younger children, which was significantly higher than that of 7.2° achieved in the older children; *p* = 0.027.

**Figure 2:**
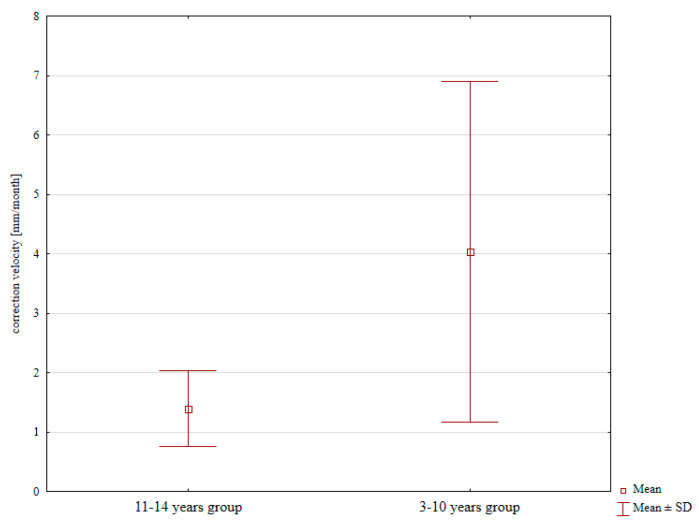
Correction velocity values depending on age.

The mean correction velocity was 1.23 mm/month in females and 2.46 mm/month in males, with the difference not significant. However, the difference in mean correction velocity between 3–10-year-old children (4.03 mm/month) and that in 11–14-year-old children (1.39 mm/month) was statistically significant; *p* = 0.031; Figure 2.

**Figure 3:**
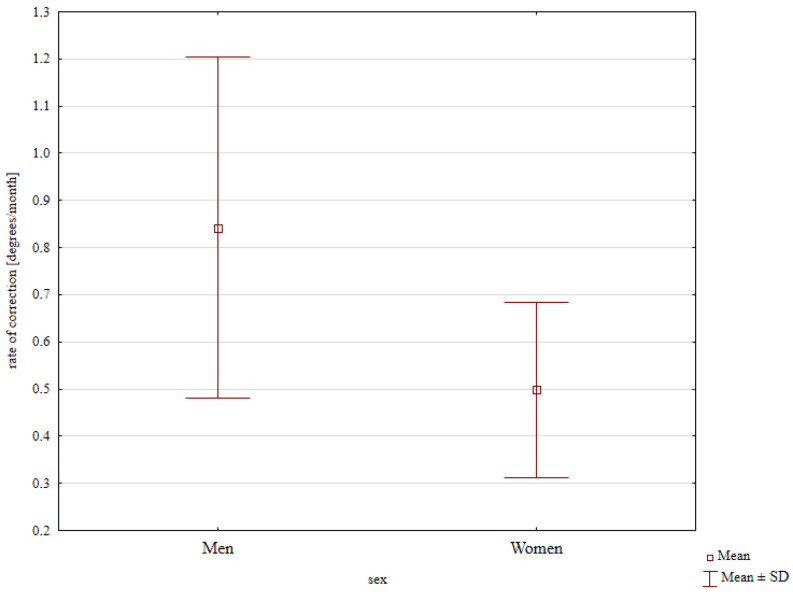
Rate of correction values depending on gender.

**Figure 4:**
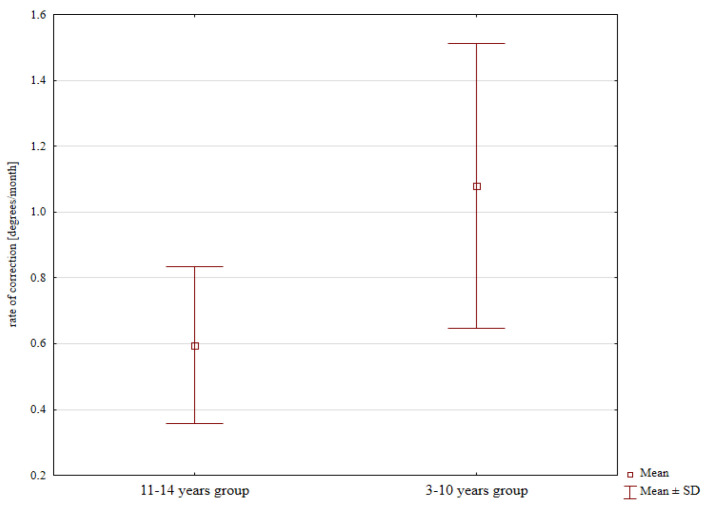
Rate of correction values depending on age.

The mean rate of correction was 0.49°/month in females and 0.89°/month in males, with the latter rate significantly greater; *p* = 0.023; Figure 3. Moreover, the difference in the mean rate of correction between the younger (1.08°/month) and the older subgroup (0.59°/month) was also significant; *p* = 0.018; Figure 4.

Complete deformity correction was observed in 85.71% of males and 72.73% of females and in 84.21% of 3–10-year-olds and 66.67% of 11–14-year-olds, with neither difference statistically significant. 

Deformity recurrence was observed in 14.29% of male patients and 36.36% of female patients, showing no significant difference between these subgroups. However, a significant difference in terms of the deformity recurrence rates was observed between the younger subgroup (66.67%) and older subgroup (only 10.53%); *p* = 0.005.

A total of 4.55% of males and 3.85% of females developed complications. Similarly, non-significant differences in complication rates were noted between the subgroup of 3–10-year-olds (5%) and 11–14-year-olds (3.57%). We observed two complications. In one case there was a loosened implant, which required revision surgery, and in the other case, there was a surgical wound infection, which was successfully treated with a 10-day course of an oral antibiotic and dressing changes. 

## 4. Discussion

Our study assessed if patient sex or age can affect various clinical or radiological parameters of hemiepiphysiodesis. We ascertained that patient age has a measurable effect on the outcomes of this procedure, whereas sex affects only some of the evaluated parameters. The age at which patients underwent hemiepiphysiodesis significantly affected the duration of surgery, amount of angular correction, correction velocity, rate of correction, and recurrence of deformity. The male and female subgroups showed divergent outcomes only in terms of deformity correction time and rate of correction. Our results partially support our research hypothesis. 

Hemiepiphysiodesis is an acknowledged, minimally invasive, effective method of treatment for genu valgum and varum deformity correction in pediatric patients [1,2,3,6,7,8,9,10,11,12,13,14,15,21,22,23]. Previous studies focused either on the clinical and radiographic outcomes of hemiepiphysiodesis [1,2,3,9,10,11,12,13,18,21,22,23,24] or on the effects of the type of implants used [6,8,20]. However, the question of a potential impact of patient age and sex on hemiepiphysiodesis outcomes was not explored.

Girls and boys develop at different rates [25,26]. Both the “growth spurt” and the end of bone growth occur at different ages in the two sexes [25,26]. In boys, the period of intensive growth appears almost 2 years later and is more intense than in girls [25,26]. These factors may have been responsible for the observed differences in hemiepiphysiodesis outcomes. However, the lack of methods for predicting the extent of angular deformity progression in children poses a challenge in determining the optimal age for a hemiepiphysiodesis procedure [14]. The age at which hemiepiphysiodesis would be the most effective has not been precisely determined [6,22]. Hemiepiphysiodesis procedures have been reported to lead to complications, such as undercorrection and overcorrection [1,4,6,7,14,22,23], which may be associated with the age at which the procedure is performed [6,7,14,22].

Theoretically, faster deformity correction and higher rates of complete deformity correction should be achieved in younger children, who grow more rapidly. On the other hand, there have been reports of deformity recurrence (rebound) following implant removal after correction had been achieved, which may adversely affect outcomes in younger children [2,6,7,22,23].

Some authors suggest leaving the implants in place until a slight overcorrection is achieved, in order to reduce the risk of deformity recurrence [1,2,6,7,14,22]; we share this view. Recurrent deformity in patients with open growth plates may be treated via another hemiepiphysiodesis procedure, whereas patients with completed growth plate fusion may require osteotomy.

There are no available studies assessing the length of hospitalization for hemiepiphysiodesis. Our study showed the length of hospital stay to be the same irrespective of the study subgroup. This may have been a result of our standard perioperative protocol and the lack of serious complications, which might have lengthened hospitalization. 

The mean surgery duration was the same in females and males. In the subgroup of 3–10-year-old children, the surgery duration was 25.62 min, which was significantly longer than that of 11–14-year-olds. This may have been due to one of two reasons. First, selecting the right plate and screw size is more challenging and, thus, may take a longer time in younger children; second, the proximal fibula in this age subgroup may obscure the optimal location for the implant in the case of lateral tibial hemiepiphysiodesis. Nonetheless, it is worth noting that the mean duration of surgery of approximately 25 min indicates that the procedure can be conducted easily and quickly. There have been no studies assessing hemiepiphysiodesis procedure duration.

Trisolino et al., who assessed 97 children following hemiepiphysiodesis, reported a deformity correction time of 14.3–24.7 months, depending on deformity etiology [1]. Dai reported a mean deformity correction time of 13.3 months in children younger than 10 years of age [4]. Kumar et al. removed implants after a mean of 10.3 months [6]. Aslani reported a mean implant indwelling time of 17 months in the 21 assessed patients [9]. The group of 48 patients evaluated by Raab et al. had their implants removed after a mean of 21.6 months [11]. Radtke noted a mean deformity correction time of 17.3 months [12]. The group assessed by Kulkarni et al. had a mean deformity correction time of 15.6 months [18]. Another group of 23 patients had a mean deformity correction time of 18.6 months [21]. Burghardt et al. removed implants after 9.5 months after surgery [22]. Another group of 26 patients had a mean deformity correction time of 22.7 months [23]. Makarewich et al. reported removing implants in 10 patients after hemiepiphysiodesis after a mean of 30.3 months postoperatively [24]. In our patient population, we observed that patient sex affected deformity correction time, with the time being longer in females; whereas patient age at the time of surgery seemed to be of no consequence for this parameter. The mean deformity correction time observed in our patient population was consistent with those reported in the literature [1,4,6,9,11,12,18,21,22,23,24]. 

Brauwer et al. reported a mean angular correction by 9 degrees with the use of stapling [8]. Raab reported a mean angular correction of 8 degrees [11]. Raab et al. suggest the use of osteotomy in deformities exceeding 12 degrees [11]. The amount of angular correction in our study varied depending on the patient age, with the patient sex having no effect on this outcome parameter. In children aged 3–10 years, we observed a greater mean angular correction in comparison with that achieved in 11–14-year-olds. This may have been due to the fact that greater baseline angular deformity prompted hemiepiphysiodesis at a younger age. The amount of angular deformity correction achieved in our study (7–10 degrees) is similar to those reported by other authors (8–9 degrees) [8,11].

Radtke reported a mean MAD correction of 5.96–10.16 mm following treatment in various patient subgroups [12]. Makarewich et al. observed a mean MAD correction of 26.1 mm [24]. In our study, neither patient sex nor age had any effect on the amount of MAD correction. However, we would like to emphasize that in the younger subgroup, the mean MAD correction was 38 mm whereas in the older subgroup it was 15.8 mm; however, this seemingly large difference was not statistically significant. The amounts of MAD correction observed in our study were slightly higher than those reported in the literature [12,24].

The patients assessed by Trisolino et al. achieved an MAD correction velocity of 0.93–1.66 mm/month [1]. Radtke reported a mean correction velocity of 0.37–0.92 mm/month in their various patient groups [12]. The population analyzed by Schagemann et al. achieved a mean correction velocity of 3.2 mm/month [19]. Our study showed no effect of patient sex on correction velocity. However, we observed a higher correction velocity in the group of 3–10-year-olds than in the older subgroup. This may have been due to the fact that younger children grow more rapidly and intensely. The correction velocity values observed in our patient population are consistent with those reported by other authors [1,12,19]. 

Dai et al. observed the rate of correction of 0.8–1.3 degrees per month in children under 10 years of age [4]. Kumar reported a mean rate of correction of 1.4 degrees per month in a group of 19 patients following eight-plate hemiepiphysiodesis [6]. Wiemann et al. noted a mean rate of correction of 10 degrees per month [17]. Another population of 24 children showed a mean correction rate of 1.5 degrees per month [18]. Schagemann reported a mean correction rate of 1 degree per month [19]. Our study demonstrated the effects of both sex and age on the rate of correction. We observed higher correction rates in males than in females and in younger children than in older children. These results may have been due to the more rapid growth in younger children and the different ages of major “growth spurts” between the two sexes. The rates of correction in our study are similar to those reported by other authors [4,6,17,18,19]. 

A study by Trisolino et al. showed complete correction in 57% of patients [1]. Dai et al. reported complete correction in 94.1% of patients up to the age of 10 years [4]. A review presented by Masquijo et al. showed a complete correction in 86–100% of patients [7]. Aslani observed complete correction in 86% of the 21 evaluated patients [9]. Kulkarni reported complete correction in 91.7% of patients [18]. In the group evaluated by Schagemann, complete correction was observed in 91% of patients [19]. Kurup reported complete correction in 77.5% patients [21]. A different population of 26 patients after hemiepiphysiodesis achieved complete correction in 76.9% of cases [23]. The proportions of complete correction achieved in our study showed no relationship with either sex or age and were similar to those reported in the literature [1,4,7,9,18,19,21,23].

Trisolino et al. observed complications in 7% of patients [1]. Dai et al. noted complications in 5% of patients [4]. In the group evaluated by Kumar, the rate of complications was 13% [6]. Aslani reported complications in 10% of the 21 evaluated patients [9]. Out of the 25 patients assessed by Ballal et al., 8% developed complications [10]. Radtke observed complications in 7.6% of patients [12]. The rate of complications reported by Wiemann et al. was 12.5% [17]. Kulkarni et al. observed complications in 8.3% of patients following hemiepiphysiodesis [18]. Schagemann reported overcorrection in 6.4% of patients [19]. In the group evaluated by Kurup et al., 47.8% of patients developed complications [21]. In another group, 7.7% of 26 patients developed complications following hemiepiphysiodesis [23]. In our patient population, neither patient age nor sex affected the observed complication rates, which were slightly lower than those reported in the literature [1,4,6,9,12,17,18,19,21,23]. Our study results support the notion that hemiepiphysiodesis is a safe method of deformity correction and has low complication rates.

Dai observed deformity recurrence in 3% of patients [4]. Kupar et al. noted no cases of deformity recurrence in the evaluated group of patients aged 4–12 years (mean age 7.8 years) [6]. Forty percent of patients assessed by Schagemann developed deformity recurrence [19]. In our patient population, patient sex showed no effect on deformity recurrence. However, 66.67% of the evaluated 3–10-year-olds developed deformity recurrence, which was a significantly higher rate than that observed in 11–14-year-olds. In the younger subgroup, implants were removed once correction was achieved; however, these patients continued to grow and if the normal, symmetrical growth plate activity was, for various reasons, disrupted, those children developed deformity recurrence. The time from implant removal to growth plate fusion in these 3–10-year-olds was longer than that in 11–14-year-olds, which provided a longer time window for deformity recurrence. The rates of deformity recurrence reported in the literature [4,6,19] are lower than those observed in our study. Children under 10 years of age seem to be at a higher risk of deformity recurrence following hemiepiphysiodesis.

Different etiologies may affect treatment outcomes and the effectiveness of hemiepiphysiodesis [1,4,5,7,11]; therefore, in our study we evaluated only patients with idiopathic deformities. A higher number of complications have been observed in patients with Blount’s disease and skeletal dysplasia [5,7]. Non-idiopathic etiology increases the risk of deformity correction failure and complications [4,7]. In our study, we excluded cases of post-traumatic deformities, post-inflammatory deformities, and deformities due to neurologic or metabolic conditions. Interestingly, the type of deformity (genu valgum vs. genu varum) has been reported to have no effect on hemiepiphysiodesis outcomes [19,21].

One of the limitations of our study was the sample size, which was not very large. This results from a limited number of patients available for analysis. Nonetheless, other authors assessed patient populations that were similar or even smaller in size [3,4,6,8,9,10,11,18,20,21,22,23,24]. Another limitation of our study is the mean follow-up period, although other studies on hemiepiphysiodesis used a follow-up of similar duration [4,9,10,18,19]. Our study was retrospective in nature, as are studies on hemiepiphysiodesis conducted by other authors [1,2,3,4,8,10,11,12,17,19,20,22,24]. One of the strengths of our study was the fact that the surgical procedures were conducted by only two experienced surgeons with the use of a single implant type in all patients. In the future, we are planning a similar analysis in a larger group of patients over a longer follow-up period.

Our study demonstrated that O-plate hemiepiphysiodesis is an effective, fast, minimally invasive, and safe treatment method for genu valgum and varum deformities in children, irrespective of sex and age.

## 5. Conclusions

Overall, patient sex had no significant effect on hemiepiphysiodesis outcomes.

Female patients showed only a longer deformity correction time and lower rate of correction in comparison with those parameters in males.

Patient age has a considerable effect on hemiepiphysiodesis outcomes.

The group of 3–10-year-olds showed longer surgery duration, a greater amount of angular correction, higher correction velocity, higher rate of correction, and higher proportion of deformity recurrence, in comparison with these parameters in 11–14-year-olds. 

## Figures and Tables

**Table 1:** Detailed assessment depending on patient gender.

Analyzed Variable	Men	Women	*p* Value
	Mean ± Standard Deviation		
Length of hospital stay (days)	3 ± 0	3 ± 0	1 *
Duration of surgery (minutes)	23.69 ± 3.44	23.8 ± 4.39	0.828 *
MAD correction amount (mm)	21.84 ± 17.18	16.72 ± 7.37	0.976 *
Amount of angular correction (degrees)	8.53 ± 2.25	6.81 ± 1.72	0.052 *
Deformity correction time (month)	11.33 ± 2.48	15.87 ± 3.81	0.013 *
Correction velocity (mm/month)	2.46 ± 2.09	1.23 ± 0.45	0.173 *
Rate of correction (degrees/month)	0.84 ± 0.36	0.49 ± 0.18	0.023 *
Complete correction (%)	85.71	72.73	0.420 **
Recurrence of deformity (%)	14.29	36.36	0.199 **
Complications (%)	4.55	3.85	0.951 **

* Mann–Whitney U Test; ** Pearson’s chi-squared test.

**Table 2:** Detailed assessment depending on patient age.

Analyzed Variable	3–10 Years Group	11–14 Years Group	*p* Value *
	Mean ± Standard Deviation		
Length of hospital stay (days)	3 ± 0	3 ± 0	1 *
Duration of surgery (minutes)	25.62 ± 4.03	22.81 ± 3.57	0.018 *
MAD correction amount (mm)	38 ± 23.76	15.8 ± 6.91	0.052 *
Amount of angular correction (degrees)	10.5 ± 3	7.2 ± 1.54	0.027 *
Deformity correction time (month)	10.12 ± 1.43	13.91 ± 3.81	0.065 *
Correction velocity (mm/month)	4.03 ± 2.86	1.39 ± 0.64	0.031 *
Rate of correction (degrees/month)	1.08 ± 0.43	0.59 ± 0.23	0.018 *
Complete correction (%)	84.21	66.67	0.348 **
Recurrence of deformity (%)	66.67	10.53	0.005 **
Complications (%)	5.0	3.57	0.609 **

* Mann–Whitney U Test; ** Pearson’s chi-squared test.

## Data Availability

The data presented in this study are available on request from the corresponding author.
